# The association of self-efficacy and coping strategies with body mass index is mediated by eating behaviors and dietary intake among young females: A structural-equation modeling approach

**DOI:** 10.1371/journal.pone.0279364

**Published:** 2023-01-27

**Authors:** Aydin Aynehchi, Sevda Saleh-Ghadimi, Parvin Dehghan

**Affiliations:** 1 Student Research committee, Tabriz University of Medical Sciences, Tabriz, Iran; 2 Clinical Research Development Unit of Tabriz Valiasr Hospital, Tabriz University of Medical Sciences, Tabriz, Iran; 3 Nutrition Research Center, Tabriz University of Medical Sciences, Tabriz, Iran; Shahrood University of Medical Sciences, ISLAMIC REPUBLIC OF IRAN

## Abstract

Globally, around three billion people are either under- or overweight. Speculating the different roles of psychological factors in body weight between over- and underweight people, it was first hypothesized that whether or not the effects of self-efficacy and coping strategies on body mass index (BMI) is different between these two groups. We secondly predicted that their association is mediated by nutritional factors. Therefore, the present cross-sectional study was conducted to model the impact of self-efficacy and coping strategies on eating behaviors, dietary intake and BMI, using structural equation modeling in two BMI groups: low-to-normal-BMI (LBMI: BMI<21.75 kg/m^2^) and normal-to-high-BMI (HBMI: 21.75 kg/m^2^ ≤ BMI). Female participants (N = 250, aged ≥18) were included using convenience sampling method and data of self-efficacy, coping strategies, eating behaviors and dietary intake were collected via questionnaires. The model fit was evaluated and confirmed by fit indices. The analysis revealed in both groups the participants tended to adopt emotion-focused coping strategy (EFCS) more than problem-focused coping strategy (PFCS) (mean score: 61.82 (7.96) vs 49.21 (6.73)). The HBMI group tended to use EFCS more than the LBMI group (P<0.001). In the LBMI group, self-efficacy, PFCS and EFCS had positive effects on BMI. Only the direct effect of self-efficacy (β = 0.314, P<0.001) and the indirect effects of PFCS and EFCS (through increasing unhealthy eating behaviors; β = 0.127, P<0.01, β = 0.095, P<0.05, respectively) were significant. In the HBMI group, self-efficacy had negative effect on BMI (both directly (β = -0.229, P<0.05) and indirectly (β = -0.103, P<0.05) by decreasing unhealthy eating behaviors). PFCS had no effect and EFCS had an indirect, positive effect on BMI (through increasing unhealthy eating behaviors, β = 0.162, P = 0.001). In conclusion, these findings suggest that improving self-efficacy and coping-strategies seems helpful to have a healthy BMI in both BMI groups and further interventions to reduce EFCS should be limited to overweight people.

## Introduction

According to a 2017 report by the World Health Organization, more than 815 million people are undernourished throughout the world and around 1.9 billion adults are overweight and obese [1]. Based on these data, a large proportion of human population is under threat, because it is generally accepted that any significant deviation from normal weight (over/underweight) has major health consequences [2]. For instance, in a well-designed population-based cohort study in 2018, Bhaskaran et al. investigated the data of about 3.6 million adults in UK and concluded that, in most cases, there was either a "U- or J-shaped" relationship between various causes of mortality and Body Mass Index (BMI) and the lowest risks were shown in people with normal BMI [3]. This is of particular importance among women, because on one hand, the international obesity rate is higher among them (15% vs. 11% in men [1]) and on the other hand, when they are underweight they are at risk of developing diseases such as breast and cervical cancer as well as reproductive problems [4, 5]. Hence, it is worthwhile to understand the interplay between potential factors that contribute to women’s weight management.

Interactive association of genetic, epigenetic and environmental factors are involved in the pathogenesis of obesity. Based on reports, estimated heritability is between 40% and 70%, as an important factor for an individual body weight [6]. Beside the inheritance, the weight of each person is the result of a balance between dietary intake and energy expenditure and is strictly controlled by physiological and psychological mechanisms [7]. For instance, it is well known that psychological distress is associated with unhealthy eating behaviors such as over/undereating and binging [8]. In addition, a vast number of environmental factors such as food security, economic status, knowledge, education, social relationships and gender discrimination can influence body weight [9]. Accordingly, the solution to overcome the obesogenic environment would be to correct the lack of accessible and immediate and healthy foods, the scarcity of safe recreational spaces in addition to environmental hazards related to low socioeconomic status, high-density communities. Moreover, everyone requires learning special abilities to overcome health-related problems and adopt a healthy eating behavior and physical activity for having a suitable BMI [10].

Concept of self-efficacy is part of Bandura’s (1989) social cognitive theory of motivation and behavior. According to this theory, self-efficacy is defined as the belief of individuals in their capability to perform a task and attain a desired outcome [11]. The effect of self-efficacy has been investigated in the management of numerous health problems such as stress [12, 13], smoking or drinking [14, 15] and adherence to medical advice [16]. The overall finding of these studies is that the more self-efficient the patients are, the more successful they are in the management of their diseases [17]. From nutritional point of view, self-efficacy accompanies healthy eating and encourages abstinence from unhealthy foods [18]. Therefore, self-efficacy may improve eating behaviors and BMI [19]. Nevertheless, further studies are needed to clarify the role of self-efficacy in this context.

Coping strategies are cognitive and behavioral efforts to reduce the perceived imbalance in a person-environment relationship that serve to control or avoid distress [20]. Women, especially in developing countries, consistently interact with social and cultural pressures [21]. Generally, a person adopts different kinds of coping strategies such as emotion-focused coping strategy (EFCS) and problem-focused coping strategy (PFCS). EFCSs are implemented to reduce emotional distress and are often less adaptive. In contrast, PFCSs are aimed to manage the situation and include problem definition, solution finding, cost-effectiveness assessment, approach selection and action and are often more appropriate than EFCSs [22]. Existing evidence indicates that girls experience severe weight-based victimization, and in response, adopt coping strategies such as binge eating and reduced physical activity or extreme food restriction and sever exercise [23, 24]. In a web-based study, inappropriate coping strategies in response to body image dissatisfaction was reported and the researchers suggested that it is necessary to help women to improve their coping behaviors [25]. Overall, understanding how women cope with environmental challenges and weight related discrepancies is helpful to develop health promotion programs.

In the current study, we aimed to evaluate the possible role of self-efficacy and coping strategies in eating behaviors and BMI of young female university students. First, direct effects of self-efficacy and coping strategies on BMI were assessed. Then, the mediating roles of eating behaviors and dietary intake were evaluated. Owing to the findings of previous studies indicating a "U- or J-shaped" association of BMI with several diseases, we hypothesized that the psychological responses of underweight people would probably be different from those with overweight and obesity; therefore, the interplay among self-efficacy, coping strategies, eating behaviors, dietary intake and BMI were compared between two BMI groups.

## Materials and methods

### Study design and participants

A cross-sectional study was conducted in Tabriz University of Medical Sciences from January to April 2020. Apparently healthy—regardless of BMI—female students (N = 250) aging ≥18 years were recruited through flyer announcements. Exclusion criteria included being pregnant or lactating, being on a diet and having any obvious psychiatric or medical condition such as anorexia or bulimia and drug abuse. Participants were divided into two BMI groups based on the midpoint of normal BMI range as the cut-off point that has been used by other researchers, as well, (low-to-normal-BMI group (LBMI): BMI<21.75 kg/m^2^, n = 125 and normal-to-high-BMI group (HBMI): BMI≥21.75 kg/m^2^, n = 125) [26, 27]. The participants entered the study after obtaining a written informed consent. All procedures performed in this study were in accordance with the 1964 Helsinki declaration and the Ethical committee of Tabriz University of Medical Sciences has approved the protocol of the study (Ethical Code: IR.TBZMED.REC.1399.752).

### Instruments and measures

#### Sample size

Sample size for each group was calculated via the "semTools" package of R software (using the "findRMSEAsamplesize" function based on a given statistical power, root mean squared error of approximation (RMSEA), alpha and the degree of freedom [obtained from the structural equation modeling (SEM) analysis of the model in STATA] of 0.80, 0.08, 0.05 and 64, respectively) in two groups that provided at least 103.5 participants per group [28]. Therefore, to account for attrition rates, 125 participants per group were recruited.

#### Anthropometric assessments

Height (using a stadiometer (Seca 206) without headdress and shoes, precision: 0.1 cm) and body weight (using a digital scale (Seca 707) with light clothing, precision: 0.1 kg) were measured and used to calculate BMI by dividing weight in kilograms by the square of height in meters.

#### Dietary intake

A three-day food record (two weekdays and one weekend day) was obtained to assess dietary intake. A dietician carefully reviewed the food records and the data of total fat (g), protein (g), carbohydrates (g) and calorie (Kcal) intake were extracted using Nutritionist IV software modified for Iranian foods.

#### Eating behavior pattern

**Fig 1:**
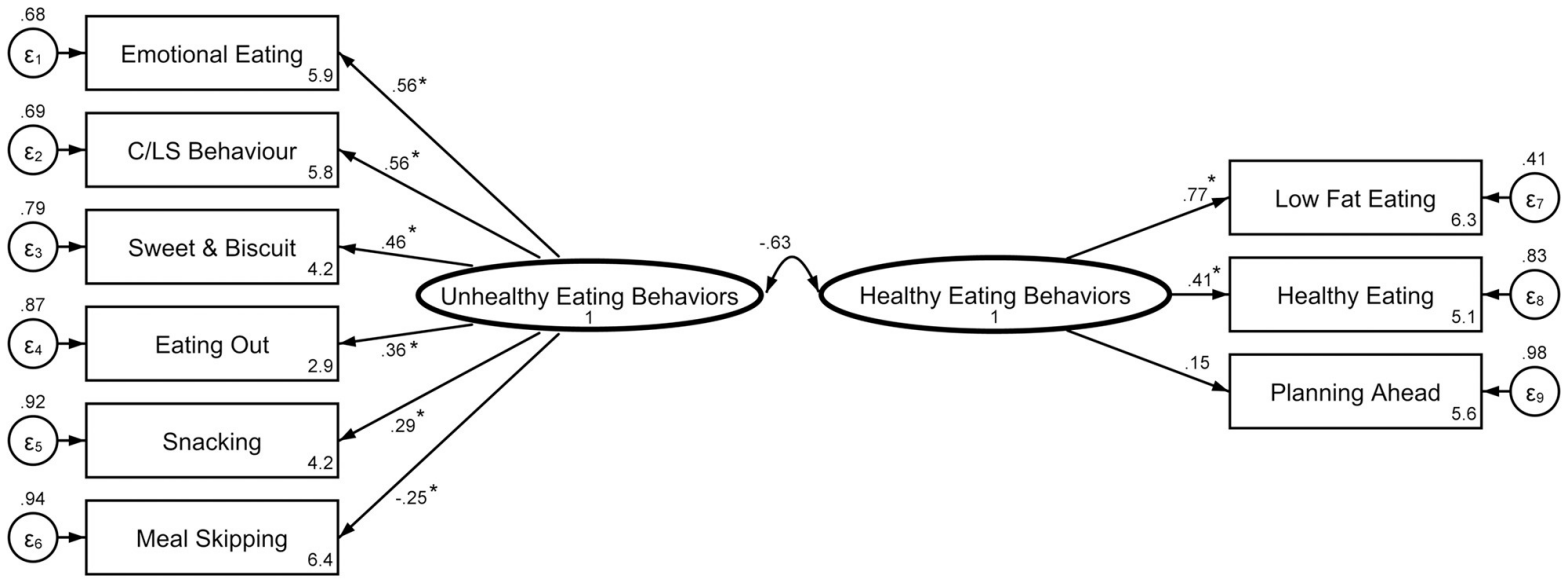
Result of confirmatory factor analysis of the eating behavior pattern questionnaire for participants. Model fit indices: χ^2^/df: 1.28; RMSEA: 0.034; CFI: 0.954; TLI: 0.936; SRMR: 0.047. ɛ1 to ɛ9 represent error variances of variables. Small numbers at the corner of the rectangular objects are standardized intercepts (the predicted value of the variables when all the predictors are zero).

Participants completed an eating behavior pattern questionnaire, which is a proper tool to evaluate the determinants of eating behavior through 48 items [29] scoring with 5-point Likert-scale ranging from strongly disagree into strongly agree. As provided in Table 1, nine categories of eating behaviors were determined by the questionnaire. It has been modified and validated locally by Dehghan et al. and Cronbach’s α coefficients for the questionnaire ranged from 0.55 to 0.78 [30]. Three factors including "low fat eating", "healthy eating" and "planning ahead" contained items describing healthier behaviors while the other six factors were generally about risky and unhealthy behaviors. Therefore, a confirmatory factor analysis (CFA) was conducted (Fig 1) in a way that these factors were collapsed into two broad "eating behaviors", the first three factors were conceptualized as the latent variable of "healthy eating behaviors" (HEB) and the remaining six as the latent variable of "unhealthy eating behaviors" (UHEB) [31]. In this study, the average inter-item correlation of each variable was 0.16 and 0.17, respectively [32] and the model fit indices were: χ^2^/df: 1.28; RMSEA: 0.034; comparative fit index (CFI): 0.954; Tucker-Lewis index (TLI): 0.936; standardized root mean square residual (SRMR): 0.047.

**Table 1:** Cronbach’s alpha and scoring for eating behavior patterns questionnaire.

Categories	Items	Scoring	α
Cultural / Lifestyle Behavior	9	9–45	0.55
Eating out	4	4–20	0.76
Emotional Eating	7	7–35	0.58
Healthy eating	4	4–20	0.54
Low fat intake	7	7–35	0.75
Meal Skipping	7	7–35	0.54
Planning Ahead	3	3–15	0.51
Snacking	3	3–15	0.71
Sweet and Biscuits	4	4–20	0.62

#### Coping strategies

Coping strategies was assessed using a standard questionnaire, Jalowiec coping scale, which assesses PFCS and EFCS, and is comprised of 39 items with responses on a 5-point Likert scale from never to always (scoring 1 to 5, respectively). PFCS is composed of 24 items scoring between 24 and 120, while EFCS is composed of 15 items scoring between 15 and 75. The questionnaire includes items such as: "exercise or did some physical activity", "tried to find out more about the problem and set up a plan of action" for PFCS and items such as: "took a drink to make yourself feel better", "took medication to make yourself feel better and ate or smoke more than usual" for EFCS. The reliability of the questionnaire has been verified in different populations [33, 34]. In the present study, the Cronbach’s coefficient alpha was 0.76 and 0.69 for PFCS and EFCS, respectively.

#### Self-efficacy

Participants filled general Sherer’s self-efficacy questionnaire [35]. The scale includes 17 items, based on a Likert scale ranging from one (strongly disagree) to five (strongly agree). The questionnaire includes items such as: "I avoid facing difficulties", "I avoid trying to learn new things when they look too difficult for me", "When I set important goals for myself I rarely achieve them". The questionnaire has a maximum score of 85, indicating the ideal self-efficacy and a minimum score of 17 as a poor self-efficacy. In 2000, Makaremi has translated it to Farsi and Cronbach’s coefficient alpha was 0.87 among Iranian college students [36]. In the present study, Cronbach’s coefficient alpha was 0.88.

### Data analysis

Statistical software including SPSS ver. 22 (SPSS Inc., Chicago, IL, USA) and STATA ver. 14 (Stata Corp, College Station, Texas USA) were used for descriptive/analytic statistics and SEM analysis, respectively. Sample size was calculated using R software ver. 4.2.0. Skewness and kurtosis in combination with Kolmogorov-Smirnov test were used to assess the normality. Independent sample t-test and Chi-squared test were performed for continuous and categorical variables, respectively. Pearson correlation was used to evaluate the relationship among study variables to establish the conditions necessary for testing mediating relationship [37]. Quadratic regression analysis was performed to test the quadratic (U-shaped) relationship between BMI and psychological variables. Using SEM, eating behavior patterns and dietary intake were examined as the mediator of the relationship between coping strategies and self-efficacy with BMI. At first, multiple imputation method, a fully conditional specification technique, was used to impute missing data. Finally, the model was examined using SEM and the fit of model was evaluated and confirmed by a set of fit indices and criteria including chi-square estimate with degrees of freedom, normed chi-square (equal to chi-square divided by its degree of freedom) (values < 5); RMSEA (values < 0.08); TLI (values > 0.9); Goodness of fit index (GFI) (values > 0.9); CFI (values >0.9) and SRMR (values < 0.08) [38].

## Results

Generally, the result of normality (skewness, kurtosis and Kolmogorov-Smirnov) test revealed that all variables were normally distributed. Skewness: age: 1.96, BMI: 0.56, EFCS: -0.05, PFCS: 0.14, self-efficacy: 0.10, protein intake: 0.62, carbohydrate intake: 0.74 and fat intake: 0.73. Kurtosis: age: 7.27, BMI: -0.07, EFCS: -0.50, PFCS: -0.23, self-efficacy: -0.39, protein intake: -0.04, carbohydrate intake: 1.06 and fat intake: 0.19. Kolmogorov-Smirnov: except age (p < 0.001) and fat intake (p = 0.01) all variables were normally distributed (p > 0.05).

All variables (EFCS, PFCS, self-efficacy, protein intake, carbohydrate intake and fat intake) were normally distributed within study groups, except age that was not normally distributed in neither of LBMI and HBMI groups based on Kolmogorov-Smirnov test (p < 0.05).

### Baseline characteristics of the participants

In total, 250 healthy female students with mean age of 22.45 years participated in this study. The basic characteristics of participants are presented in total and in BMI groups (Table 2). Most of the study participants were single (n = 198, 79.2%) and staying at home (n = 185, 74.0%). There was not a statistically significant difference between groups in terms of residence status. However, majority of participants in HBMI category were "married" (P = 0.002). The min-max of the weight, height and BMI of the participants were 42.3–86.4 kg, 149.8–182.7 cm and 16.1–33.3 kg/m^2^, in whole data, 42.3–66.9 kg, 153.0–182.7 cm and 16.1–21.7 kg/m^2^ in LBMI and 52.4–86.4 kg, 149.8–180.2 cm and 21.8–33.3 in HBMI groups, respectively. The mean (SD) of weight was 61.23 (9.24) kg in total and the body weight of the HBMI group was significantly higher than that of the LBMI group (67.56 (7.98) kg vs 54.90 (5.2) kg, P < 0.001).

**Table 2:** Selected characteristics of the participants by BMI groups (N = 250).

Variables	Total (N = 250)	BMI		p
		LBMI (< 21.75 kg/m^2^) (n = 125)	HBMI (21.75 kg/m^2^ ≤) (n = 125)	
Age (year) †	22.45 (2.49)	22.03 (2.08)	22.86 (2.80)	0.008
Marital Status ‡				
Married	52 (20.8)	16 (12.8)	36 (28.8)	0.002
Single	198 (79.2)	109 (87.2)	89 (71.2)	
Residence ‡				
Dormitory	65 (26.0)	29 (23.2)	36 (28.8)	0.313
Home	185 (74.0)	96 (76.8)	89 (71.2)	
Weight (kg) †	61.23 (9.24)	54.90 (5.20)	67.56 (7.98)	< 0.001
Height (cm) †	165.75 (5.93)	167.08 (5.70)	164.43 (5.89)	< 0.001
Body mass index (kg/m^2^) †	22.31 (3.35)	19.66 (1.49)	24.97 (2.47)	< 0.001
Coping Strategies †				
Problem-Focused	49.21 (6.73)	49.83 (5.84)	48.59 (7.49)	0.144
Emotion-Focused	61.82 (7.96)	59.66 (7.72)	63.98 (7.62)	< 0.001
Self-efficacy Score †	61.64 (8.80)	62.05 (8.40)	61.30 (8.75)	0.489

BMI: body mass index; LBMI: low-to-normal BMI group; HBMI: normal-to-high BMI group

^†^ Values are represented as mean (Standard Deviation) and p - values are based on Independent Samples T-Test.;

^‡^ Values are represented as frequency (%) and p - values are based on Chi Square test.

### Coping strategies and self-efficacy status

As presented in Table 2, in general, participants tended to adopt EFCS higher than PFCS (mean score: 61.82 (7.96) vs 49.21 (6.73)). Adopting EFCS was significantly higher in HBMI group (P < 0.001), whereas the score of PFCS and self-efficacy were not significantly different between the two groups (p>0.05).

### Dietary intake and eating behaviors of the participants

The mean daily intakes of calorie and nutrients are represented in Table 3. On average, participants consumed a diet containing 15% protein, 52% Carbohydrate and 32% fat, which consisted of exceeding the recommended intake of saturated fatty acids (SFA) (12.17% of total calorie). The mean intake of calorie and other nutrients other than SFA were significantly higher in HBMI group (p<0.05).

**Table 3:** Dietary intake and eating behavior of participants by BMI groups (N = 250).

Variables	Total	LBMI (< 21.75 kg/m^2^)	HBMI (21.75 kg/m^2^ ≤)	p†
	Mean (SD)	Mean (SD)	Mean (SD)	
Daily Dietary Intake				
Energy (Kcal)	1689.92 (437.48)	1558.85 (362.32)	1821.00 (467.33)	< 0.001
Protein (g)	63.49 (18.06)	60.58 (17.85)	66.40 (17.86)	0.01
Carbohydrate (g)	221.67 (59.24)	207.39 (49.64)	235.95 (64.59)	< 0.001
Fat (g)	60.93 (21.45)	54.09 (17.94)	67.76 (22.53)	< 0.001
SFA (%E)	12.17 (2.77)	11.96 (2.81)	12.38 (2.72)	0.238
PUFA (%E)	8.06 (2.41)	7.59 (2.50)	8.53 (2.23)	0.003
MUFA (%E)	11.86 (2.55)	11.38 (2.57)	12.34 (2.44)	0.002
Cholesterol	227.79 (118.03)	206.23 (101.78)	249.35 (129.15)	0.004
Eating Behavior Pattern Scores				
Low fat intake	26.86 (4.28)	28.38 (3.75)	25.34 (4.25)	< 0.001
Healthy eating	13.73 (2.68)	13.85 (2.66)	13.61 (2.69)	0.479
Eating out	10.39 (3.59)	10.45 (3.64)	10.33 (3.55)	0.792
Snacking	9.62 (2.28)	9.58 (2.39)	9.67 (2.18)	0.740
Sweet and Biscuits	11.63 (2.78)	10.93 (2.55)	12.34 (2.84)	< 0.001
Emotional Eating	23.58 (4.01)	22.63 (3.80)	24.52 (4.00)	< 0.001
Planning Ahead	12.19 (2.19)	12.50 (2.17)	11.88 (2.17)	0.024
Meal Skipping	23.02 (3.61)	23.54 (3.31)	22.50 (3.83)	0.023
Cultural / Lifestyle Behavior	26.76 (4.60)	25.82 (3.87)	27.70 (5.07)	0.001
Healthy Eating Behaviors	0.00 (2.66)	0.98 (2.31)	-0.98 (2.63)	< 0.001
Unhealthy Eating Behaviors	0.00 (1.80)	-0.61 (1.52)	0.61 (1.87)	< 0.001

^†^ p-value based on independent sample t-test;

Abbreviations: BMI: body mass index; LBMI: low-to-normal BMI group; HBMI: normal-to-high BMI group; SD: standard deviation; %E: mean percentage of total energy intake; SFA: saturated fatty acid; PUFA: poly-unsaturated fatty acid; MUFA: mono-unsaturated fatty acid

As shown in Table 3, the eating behavior of participants was significantly different between groups. LBMI participants had a significantly higher score of "HEB" and lower score of "UHEB" compared with HBMI group (P< 0.001) (Table 3). The score of "low fat intake", "planning ahead" and "meal skipping" was significantly higher among LBMI subjects (P< 0.05), whereas "sweet and biscuits" intake, "cultural/lifestyle behavior" and "emotional eating" were more common among HBMI group (P< 0.001).

### Relationship among study variables

Correlation among the research variables is presented in Table 4. BMI was significantly correlated with almost all of the variables except PFCS and protein intake in LBMI group. There was a positive correlation between BMI and EFCS, unhealthy eating behaviors and dietary intake, whereas BMI was negatively correlated with healthy eating behaviors in both groups. In some cases, the direction of correlations was different between groups (e.g., the correlation of BMI with self-efficiency and PFCS).

**Table 4:** Correlation among variables used in SEM modeling.

Low-to-normal BMI (< 21.75 kg/m^2^)									
	1	2	3	4	5	6	7	8	9
1. BMI									
2. Self-efficacy	0.206 *								
3. Emotion-Focused Coping	0.245**	-0.117							
4. Problem-Focused Coping	0.118	0.028	0.306**						
5. Healthy Eating Behaviors	-0.301**	0.346**	-0.223*	-0.269**					
6. Unhealthy Eating Behaviors	0.433**	-0.144	0.323**	0.327**	-0.737**				
7. Dietary Intake	0.478**	0.011	0.299**	0.173	-0.304**	0.561**			
8. Protein	0.127	0.229*	0.148	0.171	-0.065	0.218*	0.540**		
9. Carbohydrate	0.257**	0.192*	0.162	0.089	-0.028	0.261**	0.675**	0.528**	
10. Fat	0.391**	-0.034	0.274**	0.130	-0.271**	0.460**	0.973**	0.468**	0.546**
Normal-to-high-BMI (21.75 kg/m^2^ ≤)									
	1	2	3	4	5	6	7	8	9
1. BMI									
2. Self-efficacy	-0.391**								
3. Emotion-Focused Coping	0.235**	-0.304**							
4. Problem-Focused Coping	-0.212*	0.528**	0.108						
5. Healthy Eating Behaviors	-0.488**	0.454**	-0.353**	0.352**					
6. Unhealthy Eating Behaviors	0.476**	-0.344**	0.380**	-0.153	-0.777**				
7. Dietary Intake	0.528**	-0.314**	0.377**	-0.117	-0.424**	0.524**			
8. Protein	0.342**	-0.139	0.336**	0.069	-0.203*	0.301**	0.629**		
9. Carbohydrate	0.376**	-0.238**	0.312**	-0.110	-0.274**	0.343**	0.733**	0.653**	
10. Fat	0.450**	-0.288**	0.333**	-0.108	-0.355**	0.439**	0.981**	0.527**	0.623**

BMI: body mass index; SEM: structural equation modeling

* p—value is significant at the level of 0.05.

** p—value is significant at the level of 0.01.

There was a positive correlation between dietary intake and macronutrients in both groups (P < 0.01). In the LBMI group, dietary intake was correlated only with EFCS (P < 0.01) and in the HBMI group, it was correlated with both self-efficacy and EFCS (P < 0.01). However, dietary intake was not associated with PFCS in any of the groups.

In addition, to quantify the relationship between BMI and psychological variables, a quadratic regression analysis was performed. The test revealed that there were statistically significant inverted U-shaped relationships between BMI and self-efficacy (F (2, 247) = 12.167, p < 0.001) and between BMI and PFCS (F (2, 247) = 3.876, p = 0.022). BMI and EFCS had a linear relationship. The regression equations were found to be self-efficacy = -15.534 + 7.132 BMI– 0.161 (BMI^2^) and PFCS = 28.462 + 2.092 BMI– 0.051 (BMI^2^).

### Mediating effect of eating behaviors and dietary intake between coping strategies, self-efficacy and BMI

**Fig 2:**
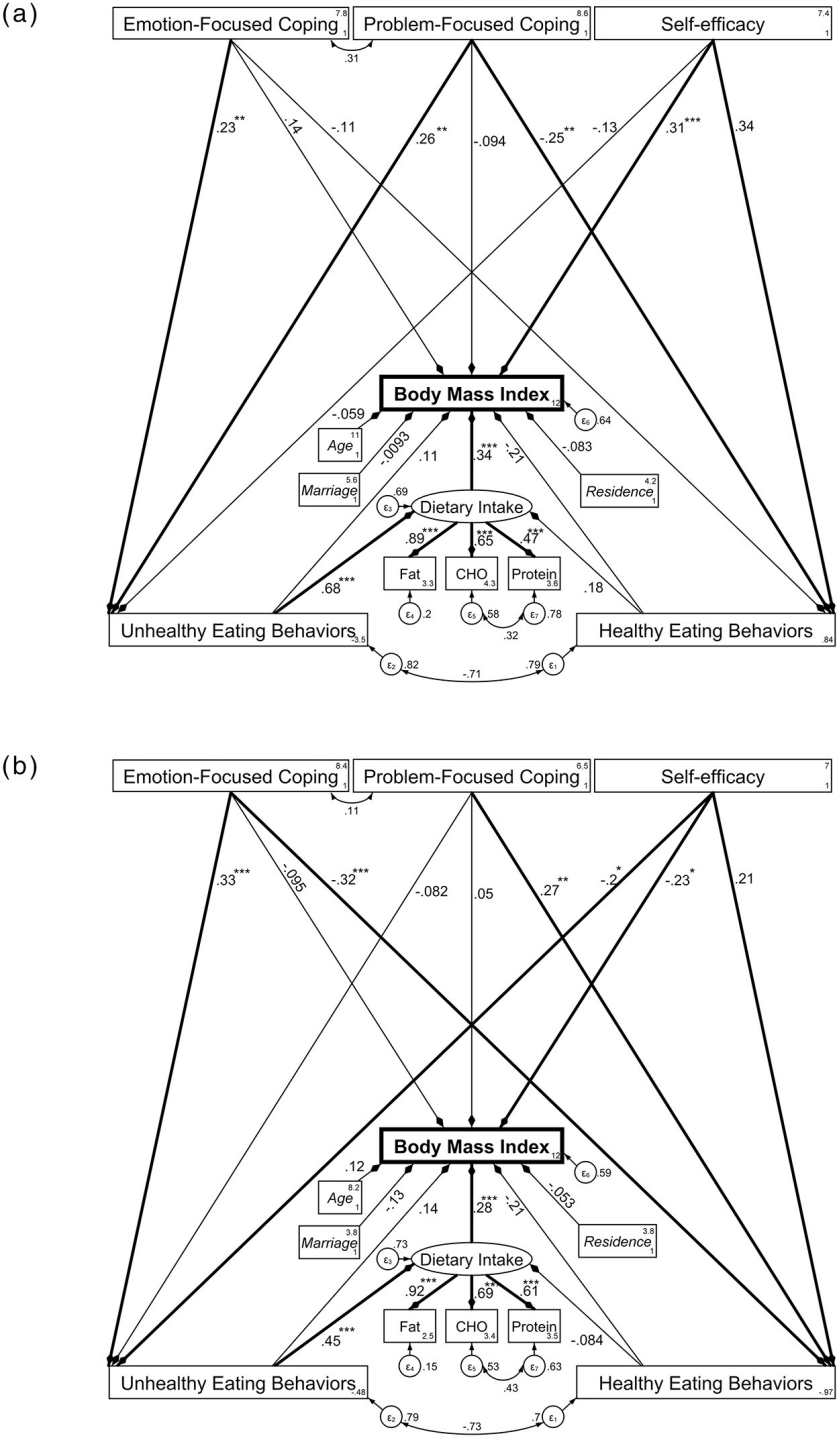
Path diagram of the studied model in two BMI categories, a: LBMI group (BMI<21.75) (N = 125) and b: HBMI group (21.75≤ BMI) (N = 125). Note: CHO: carbohydrates. Standardized coefficients are provided. Bold paths are significant (*: p<0.05; **: p<0.01 and ***: p<0.001). Potential confounding variables are illustrated in *italic* letters. Rectangles and ovals are used to represent observed vs latent variables and ɛ1 to ɛ7 represent error variances of variables. Model fit indices: χ^2^/df: 1.38; RMSEA: 0.055; CFI: 0.964; GFI: 0.888; TLI: 0.943; SRMR: 0.066.

The study model is illustrated in Fig 2 and direct, indirect and total effects are presented in Table 5. The result of SEM showed good fit to the data: χ2/df: 1.38; RMSEA: 0.055; CFI: 0.964; GFI: 0.888; TLI: 0.943; SRMR: 0.066 (Fig 2). In the LBMI group (Fig 2a), the structural model paths from self-efficacy to BMI (β = 0.31 (95% CI = 0.15–0.48), P<0.001) and also to HEB (β = 0.34 (95% CI = 0.19–0.49), P < .001), from PFCS to HEB (β = -0.25 (95% CI = -0.41- -0.08), P = .003) and also to UHEB (β = 0.26 (95% CI = 0.10–0.42), P = .002) and from EFCS to UHEB (β = 0.23 (95% CI = 0.06–0.39), P = .006) were statistically significant. None of the eating behaviors had significantly direct effects on BMI. However, the effect of HEB on BMI was insignificant (P = 0.07) and only the indirect paths from UHEB to BMI (paths from UHEB to dietary intake (β = 0.68(95% CI = 0.44–0.92), P<0.001) and from dietary intake to BMI (β = 0.34 (95% CI = 0.18–0.50), P<0.001)) were statistically significant (P < .001). None of the covariates (age, marriage and residence) had a significant effect on BMI, but their inclusion led to a more appropriate model fit. Conclusively, in the LBMI group, the effects of EFCS and PFCS on BMI were mostly mediated by UHEB (indirect effects of EFCS and PFCS on BMI: β = 0.095, P = 0.033 and β = 0.127, P = 0.006, respectively), but self-efficacy had a significantly direct effect on BMI (direct: β = 0.314, P<0.001 vs indirect: β = -0.095, P = 0.058). Finally, the SEM analysis in LBMI group revealed that higher scores of self-efficacy is directly associated with higher BMI. In addition, higher scores of coping strategies (PFCS and EFCS) indirectly resulted in higher BMI through more UHEB that increases BMI through raising dietary intake.

**Table 5:** Direct, indirect and total effects of evaluated models.

Variables	group*	Direct Effect			Indirect Effect			Total Effect		
		SPC	SE	P**	SPC	SE	P**	SPC	SE	P**
Healthy eating behaviors ←										
EFCS	1	-0.108	0.025	0.200	-	-	-	-0.108	0.025	0.200
	2	-0.318	0.029	**<0.001**	-	-	-	-0.318	0.029	**<0.001**
PFCS	1	-0.245	0.033	**0.003**	-	-	-	-0.245	0.033	**0.003**
	2	0.274	0.033	**0.003**	-	-	-	0.274	0.033	**0.003**
Self-efficacy	1	0.340	0.022	**<0.001**	-	-	-	0.340	0.022	**<0.001**
	2	0.212	0.029	**0.029**	-	-	-	0.212	0.029	**0.029**
Unhealthy eating behaviors ←										
EFCS	1	0.229	0.017	**0.008**	-	-	-	0.229	0.017	**0.008**
	2	0.327	0.022	**<0.001**	-	-	-	0.327	0.022	**<0.001**
PFCS	1	0.260	0.022	**0.002**	-	-	-	0.260	0.022	**0.002**
	2	-0.082	0.025	0.411	-	-	-	-0.082	0.025	0.411
Self-efficacy	1	-0.125	0.015	0.127	-	-	-	-0.125	0.015	0.127
	2	-0.202	0.022	0.052	-	-	-	-0.202	0.022	0.052
Dietary intake ←										
Healthy eating behaviors	1	0.183	0.032	0.202	-	-	-	0.183	0.032	0.202
	2	-0.084	0.035	0.529	-	-	-	-0.084	0.035	0.529
Unhealthy eating behaviors	1	0.678	0.074	**0.002**	-	-	-	0.678	0.074	**0.002**
	2	0.453	0.066	**0.012**	-	-	-	0.453	0.066	**0.012**
EFCS	1	-	-	-	0.136	0.004	**0.032**	0.136	0.004	**0.032**
	2	-	-	-	0.175	0.006	**0.013**	0.175	0.006	**0.013**
PFCS	1	-	-	-	0.132	0.005	**0.035**	0.132	0.005	**0.035**
	2	-	-	-	-0.060	0.006	0.320	-0.060	0.006	0.320
Self-efficacy	1	-	-	-	-0.023	0.003	0.691	-0.023	0.003	0.691
	2	-	-	-	-0.109	0.005	0.084	-0.109	0.005	0.084
BMI ←										
Healthy eating behaviors	1	-0.213	0.078	0.073	0.062	0.032	0.202	-0.151	0.079	0.212
	2	-0.211	0.114	0.084	-0.023	0.035	0.529	-0.234	0.118	0.063
Unhealthy eating behaviors	1	0.114	0.128	0.377	0.230	0.074	**0.002**	0.344	0.119	**0.004**
	2	0.140	0.156	0.236	0.126	0.066	**0.012**	0.266	0.156	**0.025**
EFCS	1	0.139	0.016	0.087	0.095	0.009	**0.033**	0.234	0.017	**0.008**
	2	-0.095	0.028	0.273	0.162	0.016	**0.001**	0.067	0.029	0.463
PFCS	1	-0.094	0.021	0.243	0.127	0.012	**0.006**	0.032	0.023	0.712
	2	0.050	0.030	0.591	-0.086	0.017	0.105	-0.036	0.033	0.714
Self-efficacy	1	0.314	0.015	**<0.001**	-0.095	0.009	0.058	0.219	0.016	**0.012**
	2	-0.229	0.026	**0.015**	-0.103	0.014	**0.041**	-0.332	0.029	**0.001**
Age	1	-0.059	0.059	0.473	-	-	-	-0.059	0.059	0.473
	2	0.120	0.066	0.109	-	-	-	0.120	0.066	0.109
Marriage	1	-0.009	0.353	0.906	-	-	-	-0.009	0.353	0.906
	2	-0.127	0.394	0.081	-	-	-	-0.127	0.394	0.081
Residence	1	-0.083	0.274	0.284	-	-	-	-0.083	0.274	0.284
	2	-0.053	0.387	0.463	-	-	-	-0.053	0.387	0.463

EFCS: emotion-focused coping strategies; PFCS: problem focused coping strategies; BMI: body mass index; SPC: standardized path coefficient; SE: standard error

*: 1: Low-to-normal BMI group and 2: Normal-to-high-BMI group

** 2-sided P-value

In HBMI group, among the psychological variables (EFCS, PFCS and self-efficacy), only the direct path between self-efficacy and BMI was statistically significant (β = -0.23 (95% CI = -0.41- -0.05), P = 0.015) (Fig 2b and Table 5). Except for the path from PFCS to UHEB, all other paths from psychological variables to HEB and UHEB were statistically significant (self-efficacy to HEB: β = 0.21 (95% CI = 0.02–0.40), P = 0.027; self-efficacy to UHEB: β = -0.20 (95% CI = -0.40- -0.00), P = 0.49; PFCS to HEB: β = 0.27 (95% CI = 0.09–0.45), P<0.01; EFCS to HEB: β = -0.32 (95% CI = -0.48- -0.16), P<0.001 and EFCS to UHEB: β = 0.33 (95% CI = 0.16–0.49), P<0.001). Neither the direct path (β = -0.21 (95% CI = -0.45–0.03), P = 0.082) nor the indirect path from HEB to BMI were significant. On the contrary, the path between UHEB and BMI was significantly mediated through dietary intake (indirect effect of UHEB on BMI: β = 0.126, P = 0.012). In HBMI group, the effect of EFCS on BMI (indirect effect: β = 0.162, P = 0.001) was mostly mediated by eating behaviors, while self-efficacy affected BMI both directly, as well as indirectly through affecting UHEB (direct: β = -0.229, P = 0.015 vs indirect: β = -0.103, P = 0.041). On the other hand, PFCS did not affect BMI significantly (total effect: β = -0.036, P = 0.714). In total, the result of SEM analysis in HBMI group showed that higher self-efficacy resulted in lower BMI through both direct and indirect path (reducing UHEB). In addition, more EFCS tends to increase BMI indirectly through increasing UHEB.

### The difference of SEM model between study groups

The difference of SEM model between study groups is presented in Table 6. Effect of different variables on eating behaviors is as follows: When comparing two groups, self-efficacy showed a similar effect on both HEB and UHEB (p = 0.418 and 0.444, respectively). In contrast, the effect of PFCS on both eating behaviors and EFCS on HEB were significantly different between groups. Considering HEB, the effects of both PFCS and EFCS were significantly different between groups (P < 0.001 and P = 0.042, respectively). Among the variables whose effect was evaluated on BMI, only the effect of self-efficacy was statistically different between groups (P<0.001). However, the effect of EFCS on BMI was marginally different between two study groups (P = 0.050).

**Table 6:** Tests for group invariance (low-to-normal vs. normal-to-high BMI groups) of parameters used in SEM modeling.

Variables	Chi-Squared	p
Healthy eating behaviors ←		
EFCS †	4.135	0.042
PFCS †	17.211	< 0.001
Self-efficacy †	0.655	0.418
Unhealthy eating behaviors ←		
EFCS †	1.620	0.203
PFCS †	7.006	0.008
Self-efficacy †	0.586	0.444
BMI ←		
Healthy eating behaviors †	0.039	0.840
Unhealthy eating behaviors †	0.005	0.940
Dietary intake ‡	0.94	0.330
EFCS †	3.838	0.050
PFCS †	1.360	0.240
Self-efficacy †	16.962	< 0.001
Age †	2.509	0.110
Marriage †	1.432	0.230
Residence †	0.021	0.880
Dietary intake ←		
Healthy eating behaviors †	1.634	0.201
Unhealthy eating behaviors †	0.854	0.360
Protein ‡	0.692	0.406
Carbohydrates ‡	1.220	0.269
Fat ‡	1.704	0.192

^†^ p-value based on Wald test;

^‡^ p-value based on Score test;

EFCS: emotion-focused coping strategies; PFCS: problem focused coping strategies; BMI: body mass index

## Discussion

The present study takes a new look at the predictors of BMI in female university students using structural equation modeling approach. The direct effect of psychological variables (self-efficacy and coping strategies) on BMI and the mediating role of eating behaviors were investigated. Regarding the fact that both low and high levels of BMIs are considered unhealthy and abnormal, and due to an inverted "U- or J-shaped" association between BMI and other diseases in the literature, we compared the relationships of the study variables between participants with low and high BMI. Therefore, three hypotheses were defined: a) more self-efficacy and PFCS and less EFCS would improve BMI, b) eating behaviors could partially mediate the relationship of self-efficacy and coping strategies with BMI, and c) the relationships among variables are different between two BMI groups (LBMI and HBMI). In this research, SEM analysis partially confirmed all these hypotheses.

First, in parallel with the aforementioned nonlinear association between BMI and many health-related parameters, similar inverted U-shaped relationships were observed between BMI and psychological variables (self-efficacy and PFCS) in this study. In addition, regarding to the fact that SEM analysis is usually performed based on the assumption of a linear association between observed and latent variables, the division of data into two BMI groups converted the U-shaped associations to linear ones and made the SEM analysis possible. Moreover, a high BMI in LBMI group and conversely, a low BMI in HBMI group were considered as favorable outcomes.

In this study, self-efficacy had a beneficial effect on the BMI of both groups. In other words, although self-efficacy and BMI were positively associated in LBMI group, they were negatively associated in HBMI group. Accordingly, in terms of self-efficacy, the first and third hypotheses were accepted. This was an important finding that was in accordance with previous studies, so that higher self-efficacy values has been associated with better health-related outcomes and quality of life [39]. Our result is also consistent with other studies reporting a negative association between self-efficacy and BMI in women [40] and university students [41]. The inverse association of self-efficacy with body weight has been observed in other populations, as well. For instance, Steele et al. [42] reported that self-efficacy was higher in normal-weight adolescents than their obese counterparts.

In this study, we observed that direct effect of self-efficacy on BMI was strongly significant; however, the indirect effect was significant only in HBMI group (through UHEB). The significant direct effect suggests that, there might be other mediators such as physical activity that are involved in this relationship [43]. The insignificance of the indirect effect in LBMI group, does not supported the second hypothesis of the mediating role of eating behaviors in this group and was probably because eating behaviors in this group is under influence of other factors such as emotional condition, economic status and nutritional knowledge [44]. While a growing body of literature has suggested the self-efficacy as a predictor for eating behaviors, it has remained controversial. In some cases, studies support the view that self-efficacy can positively influence the adherence to dietary recommendations [45] and healthy food choices even in absence of normative support [46, 47]. On the contrary, a systematic review has reported that about 34% of studies could not find a statistically significant correlation between self-efficacy and healthy eating [48]. These controversies may also be a possible result of ethnic and socioeconomic diversity of study participants, as well as application of different self-efficacy and eating behavior assessment methods among studies. Furthermore, we observed a negative association between self-efficacy and UHEB in HBMI group; since according to a report by Xazela (2021) [49] self-efficacy is related to knowledge, attitude and ability, our finding can be probably because of more nutritional knowledge among self-efficient participants in HBMI group.

In the current study, the participants mostly tended to adopt EFCS instead of PFCS. This can be explained by the findings of previous studies that there is a gender difference in the implementation of coping strategies (more EFCS are used by women) and our research was performed only in female participants [50]. In addition, this suggests that our participants often encounter stressful situations in their daily lives [51]. However, there is still some controversy surrounding the coping strategies that people use about their BMI. As reported by Strickland et al. (2007), among different coping strategies, confrontive coping (analogous to PFCS) was the only strategy that African-American women implemented to have a better BMI [52]. It seems there are different approaches among Iranian and African-American women, and suggests that Iranian women are possibly under more distress that leads them to implement EFCS more than PFCS. Since, there are data suggesting that psycho-emotional problems can result in development of weight changing effects like harmful coping strategies such as emotional eating [53]. However, psychological conditions such as depression were not evaluated in the present study.

When comparing the study groups, it was observed that EFCS was more common in the HBMI group; however, the implementation of PFCS was similar between LBMI and HBMI groups. Therefore, the third hypothesis was confirmed. Based on SEM analysis, neither PFCS nor EFCS had direct effects on BMI; however, meaningful indirect effects were observed. In detail, in LBMI group, both EFCS and PFCS were positively correlated with BMI through increasing UHEB and in HBMI group, EFCS had increasing effect on BMI through raising UHEB but PFCS did not show an ameliorating effect on BMI. Therefore, the first hypothesis was partially supported in terms of coping strategies. Observing more UHEB in response to EFCS concurs well with Kuczmarski et al. (2017), where EFCS is associated with higher energy intake from snacks [54]. In the current study, with regard to the higher prevalence of EFCS in HBMI group, it is possible that participants ate more energy from snacking in this group.

Furthermore, the positive association between EFCS and BMI in the two groups should be interpreted differently. In recent years, EFCS has been divided into 2 dimensions [55], approach-oriented EFCS and the traditional EFCS, which the former is considered as an adaptive EFCS leading to better management of a stressful situation and healthier behavior. It can be interpreted that in LBMI group, an adaptive EFCS has been implemented, which has helped the BMI reach the standard range. By contrast, it played a slightly negative role in HBMI group and was associated directly with BMI, which may be due to an implementation of maladaptive EFCS. The effect of EFCS in HBMI group is similar to previous studies but its effect in LBMI group has not properly studied and the existing evidence is not sufficient.

In this study, HEB was more pronounced in LBMI group, whereas UHEB was observed to be more dominant in HBMI group. In details, while "low fat eating", "planning ahead" and "meal skipping" were more common in LBMI group, "sweet and biscuits", "emotional eating" and "cultural/lifestyle behaviors" (refers to eating a meal with high calorie and meat such as fast foods) scored more in HBMI group. These findings were somehow consistent with previous studies implying that "emotional eating", "snacking on sweets" and "haphazard planning" had positive correlation and "low fat eating" had negative correlation with BMI [29, 56]. "Meal skipping" (a part of UHEB) was also more prevalent in LBMI group. This was because "meal skipping" include statements such as "When I am upset, I tend to stop eating." and "If I am busy, I will eat a snack instead of lunch", which can result in weight reduction and are common among university students [57]. Moreover, in the present study SEM analysis showed that only UHEB (and not HEB) was significantly associated with BMI, which could be due to the demanding life of students that force them to have more UHEB. Furthermore, considering that students are facing high levels of stress, it is assumed that they used unhealthy foods to cope with stress, which is associated with weight gain and high BMI; because, there are reports of choosing sweets, salty or high-fat foods in episodes of stressful situations [58–60]. Altogether, for a remarkable improvement in BMI of students, it seems necessary to empower them with a contribution of psychological and nutritional support.

It is noteworthy to mention that even the individuals in the high BMI range were still within the normal weight category and that the approach warrants validation by comparing normal weight individuals with obese individuals with a BMI above 25. Spinosa et al. demonstrated that psychological distress and also higher emotional eating as a maladaptive coping strategy were more prevalent among participants with higher BMI [61]. Their mean of age and BMI was 35.35 years and 26.31 kg/m^2^, respectively. Comparison of self-efficacy between normal weight (control) and obese (case) Iranian adolescents revealed that obese adolescents were less self-efficient than normal weight adolescents [62]. Also, in obese subjects a poor self-efficacy score was correlated with poor weight loss [63].

This study had some limitations. First, it was a cross-sectional study with limited sample size, which did not allow us to draw a real cause-effect relationship among variables. It is suggested to perform well-designed multi-centered studies with larger sample sizes for more comprehensive evaluation. Second, it is suggested to include stress, physical activity, nutritional knowledge and economic status. Third, in this study, the questionnaires were filled self-reportedly that could increase the probability of recall-bias. However, they have been widely used in many studies and their validity and reliability have been well established. In addition, the inclusion / exclusion criteria of health status were self-reported and not evaluated by a specialist. Therefore, we believe there might be some medical / psychological problems in some of the participants that could deviate the study results. On the contrary, using SEM analysis is one of the strength of this research that is a great and powerful tool and provides a theoretical model for complex and sophisticated hypotheses with multiple factors.

## Conclusions

Overall, the present study provided empirical evidence on the difference in the effects of coping strategies and self-efficacy on BMI between LBMI and HBMI people. In addition, compared with healthy eating behaviors, it was shown that the roles of unhealthy eating behaviors and dietary intake were more prominent as mediators in this association. In this research, improving self-efficacy and coping strategies could lead to a healthier BMI in the LBMI group, and considering that EFCS resulted in a higher UHEB in the HBMI group, interventions to reduce EFCS and also promote self-efficacy seem helpful to control overweight and obesity. Further studies seem necessary to concentrate on the role of more specific nutritional self-efficacy and coping strategies in weight management. Furthermore, as provided in this study, the researchers that focus on psychological aspects of weight management should better take into account the differences between overweight and underweight people.

## Supporting information

S1 File Data file. (XLSX)

## References

[1] World Health Organization. Driving commitment for nutrition within the UN Decade of Action on Nutrition: policy brief. World Health Organization, 2018.

[2] Bradbury KE, Cairns BJ. Understanding the relation between BMI and mortality. British Medical Journal Publishing Group; 2019. doi: 10.1136/bmj.l1219 30957772

[3] Bhaskaran K, dos-Santos-Silva I, Leon DA, Douglas IJ, Smeeth L. Association of BMI with overall and cause-specific mortality: a population-based cohort study of 3· 6 million adults in the UK. The lancet Diabetes & endocrinology. 2018;6(12):944–53. 30389323 10.1016/S2213-8587(18)30288-2 PMC6249991

[4] Charkhchi P, Schabath MB, Carlos RC. Breast, cervical, and colorectal cancer screening adherence: Effect of low body mass index in women. Journal of Women’s Health. 2020;29(7):996–1006. doi: 10.1089/jwh.2019.7739 31928405

[5] Boutari C, Pappas PD, Mintziori G, Nigdelis MP, Athanasiadis L, Goulis DG, et al. The effect of underweight on female and male reproduction. Metabolism. 2020;107:154229. doi: 10.1016/j.metabol.2020.154229 32289345

[6] Diels S, Vanden Berghe W, Van Hul W. Insights into the multifactorial causation of obesity by integrated genetic and epigenetic analysis. Obesity Reviews. 2020;21(7):e13019. doi: 10.1111/obr.13019 32170999

[7] Thom G. Weight loss maintenance: physiological, psychological and clinical perspectives: University of Glasgow; 2020.

[8] Churchill S, Jessop DC. Reflective and non‐reflective antecedents of health‐related behaviour: Exploring the relative contributions of impulsivity and implicit self‐control to the prediction of dietary behaviour. Br J Health Psychol. 2011;16(2):257–72. doi: 10.1348/135910710X498688 . 21489055

[9] Newsome FA, Gravlee CC, Cardel MI. Systemic and Environmental Contributors to Obesity Inequities in Marginalized Racial and Ethnic Groups. Nursing Clinics. 2021;56(4):619–34. doi: 10.1016/j.cnur.2021.07.003 34749900

[10] Farooqi IS. Defining the neural basis of appetite and obesity: from genes to behaviour. Clinical medicine. 2014;14(3):286. doi: 10.7861/clinmedicine.14-3-286 24889574 PMC4952542

[11] Bandura A. Human agency in social cognitive theory. Am psychol. 1989;44(9):1175. doi: 10.1037/0003-066x.44.9.1175 . 2782727

[12] Sawatzky RG, Ratner PA, Richardson CG, Washburn C, Sudmant W, Mirwaldt P. Stress and depression in students: the mediating role of stress management self-efficacy. Nurs Res. 2012;61(1):13–21. doi: 10.1097/NNR.0b013e31823b1440 . 22166906

[13] Heidary S, Heidari H, Choopani R, Sedehi M. The effect of supportive care program based on Bandura self-efficacy on stress-exacerbating and stress-relieving factors of neonatal mothers admitted to neonatal intensive care unit. Payesh (Health Monitor). 2021;20(4):451–460. 10.4103/jehp.jehp_899_22 PMC10578563 37849880

[14] Schmidt S. Predictors of Smoking Cessation: How and Under Which Conditions do Intrinsic Motivation and Self-Efficacy Predict Successful Smoking Cessation: a Longitudinal Study: University of Twente; 2021.

[15] Ponrachom C, Boonchuaythanasit K, Cardinal BJ. Drinking Refusal Self-Efficacy and Alcohol Expectancy: Changing Undergraduate Students’ Alcohol Drinking Behavior. European Journal of Molecular & Clinical Medicine. 2021;7(10):2915–21.

[16] Cardwell MS. Improving Medical Adherence in Women With Gestational Diabetes Through Self-Efficacy. Clin Diabetes. 2013;31(3):110–5. doi: 10.2337/diaclin.31.3.110

[17] Jackson T, Wang Y, Wang Y, Fan H. Self-Efficacy and Chronic Pain Outcomes: A Meta-Analytic Review. J Pain. 2014;15(8):800–14. doi: 10.1016/j.jpain.2014.05.002 . 24878675

[18] Dzielska A, Mazur J, Nałęcz H, Oblacińska A, Fijałkowska A. Importance of self-efficacy in eating behavior and physical activity change of overweight and non-overweight adolescent girls participating in healthy me: a lifestyle intervention with mobile technology. Nutrients. 2020;12(7):2128. doi: 10.3390/nu12072128 32709005 PMC7400873

[19] Annesi JJ, Gorjala S. Relations of self-regulation and self-efficacy for exercise and eating and BMI change: A field investigation. Biopsychosoc Med. 2010;4:10-. doi: 10.1186/1751-0759-4-10 20815891 PMC2941739

[20] Lazarus RS. Coping theory and research: Past, present, and future. Fifty years of the research and theory of RS Lazarus: An analysis of historical and perennial issues. 1993;55(3):366–88. doi: 10.1097/00006842-199305000-00002 . 8346332

[21] Maktabi R. Enfranchised Minors: Women as People in the Middle East after the 2011 Arab Uprisings. Laws. 2017;6(1):4.

[22] Lazarus R, Folkman S. Stress, coping and illness. In: Hs Friedman, editor. Personality and Disease. New York: John Wiley & Sons Inc.; 1985. p. 105.

[23] Puhl RM, Luedicke J. Weight-based victimization among adolescents in the school setting: Emotional reactions and coping behaviors. J Youth Adolescence. 2012;41(1):27–40. doi: 10.1007/s10964-011-9713-z WOS:000298400800003. 21918904

[24] Knol LL, Brantley C. Weight Status and Emotion-and Stress-Related Eating: Testing Constructs of the Transactional Model of Stress and Coping. American Journal of Health Education. 2021;52(3):117–26.

[25] Shah P. Relationships of weight-related dissatisfaction, body image flexibility, and coping in women. 2017.

[26] Guo Y, Zhang T, Wang Z, Yu F, Xu Q, Guo W, et al. Body mass index and mortality in chronic obstructive pulmonary disease: A dose—response meta-analysis. Medicine. 2016;95(28). doi: 10.1097/MD.0000000000004225 27428228 PMC4956822

[27] Ludolph AC, Dorst J, Dreyhaupt J, Weishaupt JH, Kassubek J, Weiland U, et al. Effect of high‐caloric nutrition on survival in amyotrophic lateral sclerosis. Annals of neurology. 2020;87(2):206–16. doi: 10.1002/ana.25661 31849093

[28] Pornprasertmanit S MP, Schoemann A, Rosseel Y, Quick C, Garnier-Villarreal M, Selig J, et al. Package ‘semtools’. 2015 Jun 29:1–46.

[29] Schlundt DG, Hargreaves MK, Buchowski MS. The eating behavior patterns questionnaire predicts dietary fat intake in African American women. J Am Diet Assoc. 2003;103(3):338–45. doi: 10.1053/jada.2003.50049 . 12616256

[30] Dehghan P, Asghari-Jafarabadi M, Salekzamani S. Validity, Reliability and Feasibility of the Eating Behavior Pattern Questionnaire (EBPQ) among Iranian Female Students. Health promotion perspectives. 2015;5(2):128. doi: 10.15171/hpp.2015.015 26290828 PMC4539050

[31] Dehghan P, Aynehchi A, Saleh-Ghadimi S, Asghari Jafarabadi M, Moslemi E. Association of self-efficacy and coping with sleep quality and disturbances with an emphasis on mediating role of eating behaviors and body mass index: A structural equation modeling approach. Current Psychology. 2021:1–11.

[32] BrckaLorenz A, Chiang Y, Nelson Laird T. Internal consistency. FSSE Psychometric Portfolio Retrieved from fsse indiana edu. 2013.

[33] Piyanee K-Y, Ornuma K, Walailak P, Chanya T, Wareerat T, Hong-Gu H. The mediating effects of coping on the stress and health relationships among nursing students: a structural equation modelling approach. J Adv Nurs. 2014;70(6):1287–98. doi: 10.1111/jan.12283 . 24236992

[34] Aziznejad P, Kashaninia Z. Effect of self-care training on applying coping strategies of adolescents. Bimon J Hormozgan Uni Med Sci. 2006;10(3):256–72.

[35] Sherer M, Maddux JE, Mercandante B, Dunn SP, Jacobs B, Rogers RW. The self-efficacy scale: Construction and validation. Psychological reports. 1982;51(2):663–71. doi: 10.2466/pr0.1982.51.2.663

[36] Makaremi A. Self-efficacy and depression among Iranian college students. Psychol Rep. 2000;86(2):386–8. doi: 10.2466/pr0.2000.86.2.386 . 10840884

[37] Baron RM, Kenny DA. The moderator—mediator variable distinction in social psychological research: Conceptual, strategic, and statistical considerations. J Pers Soc Psychol. 1986;51(6):1173. doi: 10.1037//0022-3514.51.6.1173 3806354

[38] Kline RB. Principles and practice of structural equation modeling (3. Baskı). New York, NY: Guilford. 2011.

[39] Lee MK, Oh J, editors. Health-related quality of life in older adults: Its association with health literacy, self-efficacy, social support, and health-promoting behavior. Healthcare; 2020: Multidisciplinary Digital Publishing Institute. 10.3390/healthcare8040407 PMC7712387 33081352

[40] Nasir H, Azizan A, Hamid SBA. Relationships between body mass index, exercise self-efficacy and exercise capacity among postpartum women attending Klinik Kesihatan Kepala Batas, Pulau Pinang: a cross-sectional study. Journal of Islamic, Social, Economics and Development (JISED). 2021;6(36):209–19.

[41] Demirci N, Demirci PT, Demirci E. The Effects of Eating Habits, Physical Activity, Nutrition Knowledge and Self-Efficacy Levels on Obesity. Universal Journal of Educational Research. 2018;6(7):1424–30.

[42] Steele MM, Daratha KB, Bindler RC, Power TG. The relationship between self-efficacy for behaviors that promote healthy weight and clinical indicators of adiposity in a sample of early adolescents. Health education & behavior. 2011;38(6):596–602. 21474635 10.1177/1090198110387514

[43] Ouyang Y, Luo J, Teng J, Zhang T, Wang K, Li J. Research on the Influence of Media Internalized Pressure on College Students’ Sports Participation—Chained Intermediary Analysis of Social Physique Anxiety and Weight Control Self-Efficacy. Frontiers in Psychology. 2021;12:1250. doi: 10.3389/fpsyg.2021.654690 34054659 PMC8149783

[44] Murphy SP, Rose D, Hudes M, Viteri FE. Demographic and economic factors associated with dietary quality for adults in the 1987–88 Nationwide Food Consumption Survey. J Am Diet Assoc. 1992;92(11):1352–7. 1430720

[45] Trovato GM, Martines G, Trovato F, Pace P, Pirri C, Tonzuso A, et al. Psychological determinants of dietary adherence and intervention outcome in obesity: self-efficacy, dietary and physical exercise counseling. Faseb J. 2012;26(1_MeetingAbstracts):818.9. WOS:000310711300057.22071505

[46] Hamilton K, Hagger MS. Effects of self-efficacy on healthy eating depends on normative support: a prospective study of long-haul truck drivers. International journal of behavioral medicine. 2018;25(2):265–70. doi: 10.1007/s12529-017-9685-9 28849381

[47] Zhou G, Gan Y, Hamilton K, Schwarzer R. The role of social support and self-efficacy for planning fruit and vegetable intake. Journal of nutrition education and behavior. 2017;49(2):100–6. e1. 27780668 10.1016/j.jneb.2016.09.005

[48] Guillaumie L, Godin G, Vézina-Im L-A. Psychosocial determinants of fruit and vegetable intake in adult population: a systematic review. International Journal of Behavioral Nutrition and Physical Activity. 2010;7(1):12. doi: 10.1186/1479-5868-7-12 20181070 PMC2831029

[49] Xazela N, Chinyamurindi W, Shava H. The link between self-efficacy and nutrition knowledge beliefs: findings from South Africa. African Journal of Food, Agriculture, Nutrition and Development. 2021;21(1):17330–42.

[50] Kelly MM, Tyrka AR, Price LH, Carpenter LL. Sex differences in the use of coping strategies: predictors of anxiety and depressive symptoms. Depression and anxiety. 2008;25(10):839–46. 17603810 10.1002/da.20341 PMC4469465

[51] Kamble V. Effect of Problem-Focused and Emotion-Focused Coping Strategies on Academic Stress during Examinations.

[52] Strickland OL, Giger JN, Nelson MA, Davis CM. The relationships among stress, coping, social support, and weight class in premenopausal African American women at risk for coronary heart disease. J Cardiovasc Nurs. 2007;22(4):272–8. doi: 10.1097/01.JCN.0000278964.05748.d8 WOS:000247666800004. 17589278

[53] Hemmingsson E. A new model of the role of psychological and emotional distress in promoting obesity: conceptual review with implications for treatment and prevention. Obesity Reviews. 2014;15(9):769–79. doi: 10.1111/obr.12197 24931366

[54] Kuczmarski MF, Cotugna N, Pohlig RT, Beydoun MA, Adams EL, Evans MK, et al. Snacking and diet quality are associated with the coping strategies used by a socioeconomically diverse urban cohort of African-American and white adults. Journal of the Academy of Nutrition and Dietetics. 2017;117(9):1355–65. doi: 10.1016/j.jand.2017.02.010 28365052 PMC5573625

[55] Murakami H, Yasui‐Furukori N, Otaka H, Nakayama H, Murabayashi M, Mizushiri S, et al. Coping styles associated with glucose control in individuals with type 2 diabetes mellitus. Journal of Diabetes Investigation. 2020. doi: 10.1111/jdi.13225 32017452 PMC7477505

[56] Pickett S, McCoy TP, Odetola L. The influence of chronic stress and emotions on eating behavior patterns and weight among young African American women. Western journal of nursing research. 2020;42(11):894–902. doi: 10.1177/0193945919897541 31941424

[57] Yamamoto R, Tomi R, Shinzawa M, Yoshimura R, Ozaki S, Nakanishi K, et al. Associations of skipping breakfast, lunch, and dinner with weight gain and overweight/obesity in university students: a retrospective cohort study. Nutrients. 2021;13(1):271. doi: 10.3390/nu13010271 33477859 PMC7832851

[58] Farhangi MA, Dehghan P, Jahangiry L. Mental health problems in relation to eating behavior patterns, nutrient intakes and health related quality of life among Iranian female adolescents. Plos one. 2018;13(4). 10.1371/journal.pone.0195669 PMC5922554 29702683

[59] Saleh-Ghadimi S, Dehghan P, Farhangi MA, Asghari-Jafarabadi M, Jafari-Vayghan H. Could emotional eating act as a mediator between sleep quality and food intake in female students? BioPsychoSocial Medicine. 2019;13(1):15. doi: 10.1186/s13030-019-0154-3 31236132 PMC6580454

[60] Hsu T, Raposa EB. Effects of stress on eating behaviours in adolescents: a daily diary investigation. Psychology & Health. 2020:1–16. doi: 10.1080/08870446.2020.1766041 32419507

[61] Spinosa J, Christiansen P, Dickson JM, Lorenzetti V, Hardman CA. From socioeconomic disadvantage to obesity: the mediating role of psychological distress and emotional eating. Obesity. 2019;27(4):559–64. doi: 10.1002/oby.22402 30821100 PMC6593860

[62] Miri SF, Lin C-L, Irandoost K, Rezazadeh A, Pakpour HA A. Health related quality of life and Weight Self-Efficacy of Life style among normal-weight, overweight and obese Iranian adolescents: A case control study. International Journal of Pediatrics. 2017;5(11).

[63] Bas M, Donmez S. Self-efficacy and restrained eating in relation to weight loss among overweight men and women in Turkey. Appetite. 2009;52(1):209–16. doi: 10.1016/j.appet.2008.09.017 18929608

